# Association of the risk factor *UNC13A* with survival and upper motor neuron involvement in amyotrophic lateral sclerosis

**DOI:** 10.3389/fnagi.2023.1067954

**Published:** 2023-02-01

**Authors:** Arianna Manini, Valeria Casiraghi, Alberto Brusati, Alessio Maranzano, Francesco Gentile, Eleonora Colombo, Ruggero Bonetti, Silvia Peverelli, Sabrina Invernizzi, Davide Gentilini, Stefano Messina, Federico Verde, Barbara Poletti, Isabella Fogh, Claudia Morelli, Vincenzo Silani, Antonia Ratti, Nicola Ticozzi

**Affiliations:** ^1^Department of Neurology and Laboratory of Neuroscience, IRCCS Istituto Auxologico Italiano, Milan, Italy; ^2^Neurology Residency Program, Università degli Studi di Milano, Milan, Italy; ^3^Department of Medical Biotechnology and Molecular Medicine, Università degli Studi di Milano, Milan, Italy; ^4^Department of Brain and Behavioral Sciences, Università degli Studi di Pavia, Pavia, Italy; ^5^Bioinformatics and Statistical Genomics Unit, IRCCS Istituto Auxologico Italiano, Milan, Italy; ^6^Department of Pathophysiology and Transplantation, 'Dino Ferrari' Center, Università degli Studi di Milano, Milan, Italy; ^7^Maurice Wohl Clinical Neuroscience Institute, Institute of Psychiatry, Psychology and Neuroscience, King’s College, London, United Kingdom

**Keywords:** amyotrophic lateral sclerosis, UNC13A, alleles, genotype, motor neurons, behavioral symptoms

## Abstract

**Background:**

The *UNC13A* gene is an established susceptibility locus for amyotrophic lateral sclerosis (ALS) and a determinant of shorter survival after disease onset, with up to 33.0 months difference in life expectancy for carriers of the rs12608932 risk genotype. However, its overall effect on other clinical features and ALS phenotypic variability is controversial.

**Methods:**

Genotype data of the *UNC13A* rs12608932 SNP (A–major allele; C–minor allele) was obtained from a cohort of 972 ALS patients. Demographic and clinical variables were collected, including cognitive and behavioral profiles, evaluated through the Edinburgh Cognitive and Behavioral ALS Screen (ECAS) – Italian version and the Frontal Behavioral Inventory (FBI); upper and lower motor neuron involvement, assessed by the Penn Upper Motor Neuron Score (PUMNS) and the Lower Motor Neuron Score (LMNS)/Medical Research Council (MRC) scores, respectively; the ALS Functional Rating Scale Revised (ALSFRS-R) score at evaluation and progression rate; age and site of onset; survival. The comparison between the three rs12608932 genotypes (AA, AC, and CC) was performed using the additive, dominant, and recessive genetic models.

**Results:**

The rs12608932 minor allele frequency was 0.31 in our ALS cohort, in comparison to 0.33–0.41 reported in other Caucasian ALS populations. Carriers of at least one minor C allele (AC + CC genotypes) had a shorter median survival than patients with the wild-type AA genotype (−11.7 months, *p* = 0.013), even after adjusting for age and site of onset, *C9orf72* mutational status and gender. Patients harboring at least one major A allele (AA + AC genotypes) and particularly those with the wild-type AA genotype showed a significantly higher PUMNS compared to CC carriers (*p* = 0.015 and *p_adj_* = 0.037, respectively), thus indicating a more severe upper motor neuron involvement. Our analysis did not detect significant associations with all the other clinical parameters considered.

**Conclusion:**

Overall, our findings confirm the role of *UNC13A* as a determinant of survival in ALS patients and show the association of this locus also with upper motor neuron involvement.

## 1. Introduction

Amyotrophic lateral sclerosis (ALS) is a rapidly progressive motor neuron disease (MND), which usually occurs in the sixth or seventh decades, and leads to death within 3–5 years from symptoms onset (Talbott et al., 2016). Over the last years, ALS was found to be part of a spectrum disorder defined as MND-frontotemporal dementia (FTD) continuum (Burrell et al., 2016). Indeed, 5–10% of ALS patients are diagnosed with FTD and up to 50% of ALS patients develop cognitive decline or behavioral dysfunction, albeit not fulfilling the diagnostic criteria for FTD (Burrell et al., 2016). Similarly, a significant proportion of FTD patients shows signs of motor neuron involvement.

While most ALS cases are sporadic (sporadic ALS; SALS), approximately 10% of patients show a family history (familial ALS; FALS) with mutations in more than 30 genes identified so far (Goutman et al., 2022). ALS-associated causative genes account for 60–80% FALS and 10% SALS cases (Renton et al., 2014). However, the most recurring genetic defects are found in only four genes, and are represented by hexanucleotide repeat expansions in *C9orf72*, followed by mutations in *SOD1*, *TARDBP*, and *FUS*.

In line with a multifactorial view of ALS genetic architecture in sporadic cases, large international efforts have been conducted to identify susceptibility loci by genome-wide association studies (GWAS; Renton et al., 2014). Historically, in 2009 van Es and colleagues first identified a significant association peak, mapping at chromosome 19p13.3, within the intron 20–21 of the Unc-13 Homolog A (*UNC13A*) gene (van Es et al., 2009). Only one common single nucleotide polymorphism (SNP), namely rs12608932, reached genome-wide significance (*p* = 2.53 × 10^−14^) in a two-stage combined analysis for a total of 4,855 ALS patients and 14,953 control subjects (van Es et al., 2009). Subsequent replication studies and colocalization analyses across neurodegenerative diseases have not only confirmed the association of this locus with ALS susceptibility (van Rheenen et al., 2016), but also revealed that *UNC13A* is a shared susceptibility locus for ALS and FTD (van Rheenen et al., 2016; Karch et al., 2018), especially in presence of TDP-43 proteinopathy (Diekstra et al., 2014).

Patients homozygous for the minor C allele of the *UNC13A* rs12608932 SNP show a reduction of survival of 5.0–33.0 months compared to the other genotypes (Diekstra et al., 2012; Chiò et al., 2013; Vidal-Taboada et al., 2015; van Rheenen et al., 2016; Yang et al., 2019; Tan et al., 2020). Furthermore, the minor allele has been associated, although not consistently, with higher age at onset, more frequent bulbar onset and reduced forced vital capacity (Diekstra et al., 2012; The ALSGEN Consortium, 2013; Chen et al., 2014; Vidal-Taboada et al., 2015; Tan et al., 2020). Only a limited number of studies have investigated the relationship of *UNC13A* with behavioral and cognitive features of ALS patients, highlighting an association of the minor C allele with imaging, neuropsychological and pathological markers of FTD in ALS, as well as with higher rates of reported disinhibition and behavioral impairment (Placek et al., 2019; Tan et al., 2020).

In this scenario, we combined genotype data of rs12608932 and a broad variety of clinical variables, including age and site of onset, survival, upper (UMN) and lower motor neuron (LMN) signs, functional status, disease progression, cognitive dysfunction and behavioral symptoms within a large cohort of 972 Italian ALS patients, to explore the contribution of the *UNC13A* locus to ALS phenotypic variability.

## 2. Materials and methods

### 2.1. Patients’ cohort and clinical evaluation

A total of 972 patients, affected by ALS and other motor neuron diseases (primary lateral sclerosis, PLS and progressive muscular atrophy, PMA) according to the El Escorial revised criteria (Brooks et al., 2000), were enrolled at IRCCS Istituto Auxologico Italiano between 2013 and 2022. The Ethics Committee of IRCCS Istituto Auxologico Italiano approved the study (2021_05_18). Written informed consent for employing pseudo-anonymized clinical data for research purposes was obtained from all patients at the time of evaluation. The study was conducted in accordance with the principles of the Declaration of Helsinki.

We recorded the following demographic and clinical variables: gender, age at onset and at diagnosis, site of onset, clinical phenotype, ALS Functional Rating Scale – Revised version (ALSFRS-R) score at evaluation (Cedarbaum et al., 1999), and progression rate [calculated using the formula (48 – ALSFRS-R score)/disease duration at evaluation expressed in months].

The burden of UMN signs was explored with the Penn Upper Motor Neuron Score (PUMNS; Quinn et al., 2020), while involvement of LMN was measured with a modified version of the LMN score (LMNS; Devine et al., 2016), and the Medical Research Council (MRC) muscle scale, as previously described (Maranzano et al., 2022).

We assessed the prevalence of cognitive impairment by administering the Edinburgh Cognitive and Behavioural ALS Screen (ECAS) – Italian version to a subset of 254 patients (Poletti et al., 2016). The ECAS total score is composed of subdomains investigating the ALS-specific cognitive decline (language, verbal fluency, and executive functions; ALS-specific score) and others which explore memory and visuospatial functions (ALS non-specific score). Based on the performance at ECAS and according to the Strong revised criteria, patients were classified as cognitively normal (ALScn), behaviorally impaired (ALSbi), cognitively impaired (ALSci), or both cognitively and behaviorally impaired (ALScbi; Strong et al., 2017).

In a subset of 203 patients, the presence of behavioral symptoms was explored using the ECAS Carer Interview and the Frontal Behavioral Inventory (FBI; Alberici et al., 2007). In addition to the total ECAS Carer Interview score (range 0–10), we recorded also the number of behavioral symptoms for each patient (disinhibition, apathy/inertia, loss of sympathy/empathy, perseverative/stereotyped/compulsive/ritualistic behavior and hyperorality/altered food preferences; range 0–5). As regards FBI, from 0 to 3 points can be attributed to each of 24 items (total score 0–72), which investigate both negative (FBI-A) and positive/disinhibited behaviors (FBI-B).

We employed the diagnostic criteria for behavioral variant FTD (bvFTD) and primary progressive aphasia (PPA) to define the presence of ALS/FTD (Gorno-Tempini et al., 2011; Rascovsky et al., 2011).

### 2.2. Genetic analyses and *UNC13A* and SNP genotyping

All patients included in the study were screened for the hexanucleotide repeat expansion in *C9orf72* and for mutations in *SOD1* (all 5 exons), *TARDBP* (exon 6) and *FUS* (exons 5, 6, 13, 14 and 15), as previously described (Corrado et al., 2009, 2010; Ratti et al., 2022).

For 865 ALS patients, *UNC13A* rs12608932 genotyping was performed with the Illumina SNP arrays as previously described (Manini et al., 2022). For the remaining 107 patients, allele-specific polymerase chain reaction (PCR) was performed using 2 primers to amplify the whole region (forward: GGGGCAGCTTACATCATCCAT; reverse: GGATGTATAGGCAGATGGACA) and 2 allele-specific primers (reference: CCACCCATCAATTTATCCAA; alternative: ACAGACGAAAAATGGATGGG). Amplicons were then resolved on 3% agarose gel to discriminate the presence of a 273-bp band (allele C) and a 178-bp band (allele A).

### 2.3. Statistical analyses

Statistical analyses were performed with the IBM Statistical Package for the Social Sciences (SPSS) version 26. Values were reported as medians and interquartile range (IQR) for not normally distributed quantitative variables, or frequencies (%) for categorical variables. We compared variables across the three rs12608932 genotypes by employing three different genetic models: additive (CC vs. AC vs. AA), dominant [(CC + CA) vs. AA], and recessive [CC vs. (AA + AC)], where A and C are the major and minor alleles, respectively. Chi-square or Fisher exact tests were used to compare categorical variables, as appropriate. The non-parametric Kruskal–Wallis one-way analysis of variance (ANOVA) was run to compare quantitative variables between genotypes, since their distribution was not similar for all groups, as assessed by visual inspection of boxplots, and normality assumptions for parametric tests were not met according to the Kolmogorov–Smirnov normality test. When possible, *post hoc* analysis was performed to compare subgroups, including pairwise comparisons using the z-test of two proportions and the (Dunn, 1961) procedure with a Bonferroni correction for multiple testing. The impact of the rs12608932 SNP on survival was assessed by the univariate Kaplan–Meier survival analysis with a log-rank comparison test and multivariate Cox regression model analysis, using gender, age at onset, and site of onset as covariates. Censoring was applied for patients alive at last follow-up. *p* values <0.05 were considered statistically significant. In the Kaplan–Meier survival analysis, log-rank pairwise comparisons were run to determine which genotypes had different survival distributions under the additive model. A Bonferroni correction was made with statistical significance accepted at the *p* < 0.0167 level. Pairwise deletion was used to handle missing data.

## 3. Results

We genotyped the *UNC13A* rs12608932 SNP in a cohort of 972 Italian ALS patients, with a prevalence of males (n = 616, 63.4%) over females (n = 356, 36.6%; Table 1). Only 44 patients (4.8%) had a positive family history for ALS (FALS), while most cases were sporadic (SALS). The *C9orf72* repeat expansion was present in 31 patients (7 FALS, 24 SALS), whereas 10 had a mutation in *TARDBP* (4 FALS, 6 SALS), 3 in *FUS* (all SALS) and 1 in *SOD1* (FALS). We were able to retrieve the age at onset of 887 patients (median 61.6 years, IQR 52.7–69.6), the survival after disease onset of 886 (median 25.9 months, IQR 14.0–45.7), and the site of onset of 903, which was bulbar in 224 cases (24.8%) and spinal in the remaining 679 (75.2%). The median ALSFRS-R score, available for 516 patients, was 40 (IQR 35–43). Median PUMNS, recorded for 767 patients, was 9 (IQR 4–15). Amongst the 765 patients for whom the LMNS was available, median score was 2 (IQR 2–6). The median MRC total score, collected for 597 patients, was 54 (IQR 47–58). ECAS and FBI were performed in 254 and 203 patients, respectively. Concerning the ECAS, the median total score was 105 (IQR 90–114), the median ALS-specific score was 78 (IQR 67–85), and the median ALS non-specific score was 27 (IQR 24–30). Based on the performance at ECAS and according to the Strong revised criteria, 112 out of 254 patients (44.1%) could be classified as ALScn, 45 (17.7%) as ALSbi, 66 (26.0%) as ALSci, and 32 (12.6%) as ALScbi. As regards FBI, the median total score was 2 (IQR 0–5), with a median A score of 1 (IQR 0–4), and a median B score of 0 (IQR 0–2). According to the Rascovsky and Gorno-Tempini criteria for bvFTD and PPA, respectively, 26 (2.7%) of our patients were affected by ALS/FTD. All the demographic and clinical features of the cohort are reported in Table 1.

**Table 1:** Demographic and clinical features and genotype data of the ALS cohort.

Variable	N° patients (frequency)	Median (IQR)
Sex	972	
Male	616 (63.4%)	
Female	356 (36.6%)	
ALS family history	924	
FALS	44 (4.8%)	
SALS	880 (95.2%)	
Mutations in the four main ALS genes		
*C9orf72*	31 (3.2%)	
*SOD1*	1 (0.1%)	
*TARDBP*	10 (1.0%)	
*FUS*	3 (0.3%)	
Age at onset (years)	887	61.6 (52.7–69.6)
Survival (months)	886	25.9 (14.0–45.7)
Site of onset	903	
Bulbar	224 (24.8%)	
Spinal	679 (75.2%)	
ALSFRS-R	516	40 (35–43)
PUMNS	767	9 (4–15)
MRC total score	597	54 (47–58)
LMNS	765	4 (2–6)
ECAS total score	254	105 (90–114)
ECAS ALS-specific score	254	78 (67–85)
ECAS ALS non-specific	254	27 (24–30)
Cognitive phenotype (Strong revised criteria)	254	
ALScn	112 (44.1%)	
ALSbi	45 (17.7%)	
ALSci	66 (26.0%)	
ALScbi	32 (12.6%)	
FBI total score	203	2 (0–5)
FBI A score	203	1 (0–4)
FBI B score	203	0 (0–2)
N° of ALS/FTD patients	26 (2.7%)	
rs12608932 genotype	972	
AA	487 (50.1%)	
AC	376 (38.7%)	
CC	109 (11.2%)	

N°: number; IQR: interquartile range; ALS: amyotrophic lateral sclerosis; FALS: familial ALS; SALS: sporadic ALS; ECAS: Edinburgh Cognitive and Behavioral ALS Screen; FBI: Frontal Behavioural Inventory; ALS/FTD: amyotrophic lateral sclerosis – frontotemporal dementia; ALSFRS-R: ALS Functional Rating Scale Revised; PUMNS: Penn Upper Motor Neuron Score; MRC: Medical Research Council; LMNS: lower motor neuron score; ALScn: cognitively normal ALS; ALSbi: ALS with behavioural impairment; ALSci: ALS with cognitive impairment; ALScbi: ALS with cognitive and behavioural impairment.

The minor allele frequency (MAF) of the rs12608932 SNP C within our cohort was 0.31, in line with the MAF value reported in control subjects of Caucasian origin (0.29–0.36) and slightly lower compared to non-Caucasian ALS populations (0.33–0.41; Yang et al., 2019). The prevalence of the different genotypes was 50.1% (*n* = 487) for AA, 38.7% (*n* = 376) for AC, and 11.2% (*n* = 109) for CC (Table 1).

**Figure 1:**
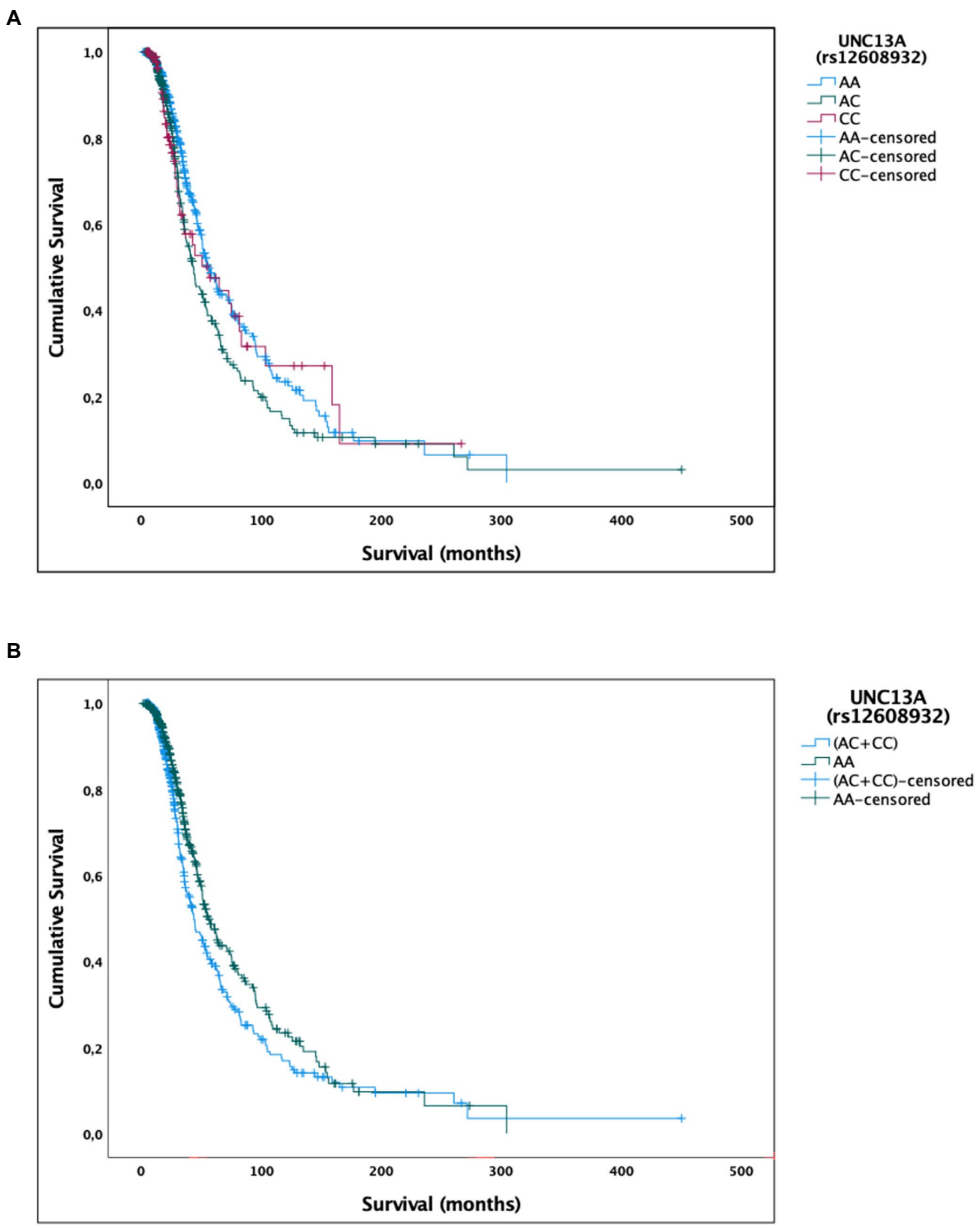
Univariate Kaplan–Meier survival analysis for the *UNC13A* rs12608932 SNP. **(A)** Additive model. **(B)** Dominant model.

We found an association between rs12608932 and median survival after disease onset under the additive and dominant models. According to the additive model, we found that the median survival was 55.4 (95% CI, 47.9–63.0) in AA, 43.5 (36.1–50.7) in AC and 56.0 months (26.7–85.3) in CC patients (*p* = 0.025; Table 2; Figure 1A). *Post hoc* analysis revealed a statistically significant difference in survival distributions between the AC vs. AA carriers [χ^2^(1) = 7.407, *p* = 0.006], but not between the CC vs. AA carriers [χ^2^(1) = 0.335, *p* = 0.563], nor between the AC vs. CC carriers [χ^2^(1) = 0.906, *p* = 0.341]. The percentage of censored cases was similar between subjects harboring the AA and CC genotypes (60.9 and 61.5%, respectively), but quite lower in the AC carriers (51.9%).

**Table 2:** Comparison of median survival time, and associated statistics, between carriers of the [AA vs. AC vs. CC] genotypes (additive model), [AA vs. (AC + CC)] genotypes (dominant model), and [(AA + AC) vs. CC] genotypes (recessive model) of the *UNC13A* rs12608932 SNP.

	Median				
	95% CI				
	Estimate	SE	Lower bound	Upper bound	*p* value
*UNC13A* (rs12608932)					
AA	55.4	3.9	47.9	63.0	
AC	43.5	3.7	36.1	50.7	
CC	56.0	14.9	26.7	85.3	
					**0.025**
*UNC13A* (rs12608932)					
AA	55.4	3.9	47.9	63.0	
(AC + CC)	43.7	3.8	36.3	51.1	
					**0.013**
*UNC13A* (rs12608932)					
(AA + AC)	51.0	2.7	45.7	56.2	
CC	56.0	14.9	26.7	85.3	
					0.915

Values are reported in months. *p* values < 0.05 are reported in bold. SNP: single nucleotide polymorphism; SE: standard error; CI: confidence interval.

As regards the dominant model, the median survival of patients harboring the CC and AC genotypes (43.7 months, 95% CI, 36.3–51.1) was significantly reduced compared to the AA carriers [55.4 months, 95% CI, 47.9–63.0 months; χ^2^(2) = 6.233, *p* = 0.013; Table 2; Figure 1B]. A similar percentage of censored cases was present in AA carriers (60.9%) and (CC + AC) genotypes (54.0%).

No statistically significant difference in median survival time was instead detected when considering the recessive model [CC vs. (AA + AC) carriers; Table 2].

A multivariate Cox regression model, including the additive model for the rs12608932 SNP, age at onset, *C9orf72* mutational status, gender, and site of onset, significantly predicted survival [χ^2^(5) = 101.786, *p* < 0.001]. Among covariates, only the rs12608932 genotypes under the additive model [B = 0.219, Exp(B) = 1.245, 95% CI = 1.069–1.450, *p* = 0.005], the age at onset [B = 0.043, Exp(B) = 1.044, 95% CI = 1.034–1.054, *p* < 0.001], and the presence of a pathological repeat expansion in *C9orf72* [B = −0.810, Exp(B) = 0.445, 95% CI = 0.277–0.716, *p* < 0.001] correlated with survival. Similarly, a Cox regression model, including the same covariates, but replacing the additive model for rs12608932 with the dominant one, significantly predicted survival [χ^2^(5) = 104.918, *p* < 0.001]. Again, the only covariates significantly associated to survival were the rs12608932 genotypes under the dominant model [B = 0.335, Exp(B) = 1.398, 95% CI = 1.135–1.721, *p* = 0.002], the age at onset [B = 0.042, Exp(B) = 1.043, 95% CI = 1.034–1.053, *p* < 0.001], and the presence of a pathological repeat expansion in *C9orf72* [B = −0.784, Exp(B) = 0.457, 95% CI = 0.284–0.735, *p* = 0.001]. Regression coefficients and standard errors are reported in Supplementary Table 1.

**Figure 2:**
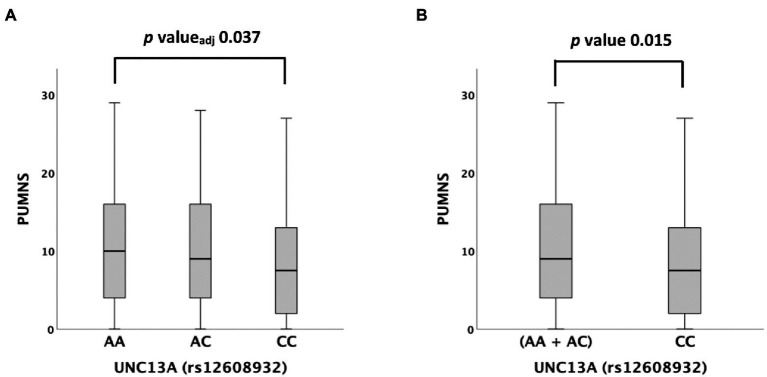
Distribution of PUMNS amongst *UNC13A* rs12608932 genotypes according to the Kruskal–Wallis one-way analysis of variance for independent samples. **(A)** Additive model. **(B)** Recessive model. For each group, the bold line shows the median, the gray box includes the middle 50% of the data and whiskers show the minimum and maximum values. Empty circles represent outliers.

We then tested possible associations with different clinical variables and scales. Among them, rs12608932 was significantly associated with the PUMNS under the additive [median value 10 (IQR 4–16) in AA vs. 9 (IQR 4–16) in AC vs. 8 (2–13) in CC, *p* = 0.044] and recessive models [median value 8 (2–13) in CC vs. 9 (IQR 4–16) in (AA + AC), *p* = 0.015; Supplementary Table 2; Figure 2]. A *post hoc* analysis showed that the PUMNS was significantly lower in CC carriers compared to AA carriers (*p* = 0.012, *p*_adjusted_ = 0.037; Figure 2A), whereas no significant difference subsisted between the AC and AA genotypes (*p* = 0.579, *p*_adjusted_ = 1.000) nor between the CC and AC genotypes after correction for multiple tests (*p* = 0.036, *p*_adjusted_ = 0.108).

No significant association was found between rs12608932 genotypes and several clinical features and parameters, including age or site of onset; ALSFRS-R and progression rate; LMNS and MRC total score; ECAS scores (total, ALS-specific and ALS non-specific); FBI scores (total, A and B); ECAS carer interview total score and number of symptoms recorded; distribution of the four ALS types according to the Strong criteria (ALScn, ALSci, ALSbi and ALScbi); prevalence of cognitively impaired vs. unimpaired patients [i.e., (ALSci + ALScbi) vs. (ALScn + ALSbi)] and of behaviorally impaired vs. unimpaired patients [i.e., (ALSbi + ALScbi) vs. (ALScn + ALSci)]; presence of ALS/FTD.

## 4. Discussion

UNC13A protein is widely expressed in both the central and the peripheral nervous system and is localized at the presynaptic membrane, where it controls the release of hormones, peptides, and neurotransmitters, including glutamate (Aravamudan et al., 1999; Augustin et al., 1999). UNC13A is involved in the priming of vesicles at the presynaptic membrane, which precedes their exocytotic fusion and content release in the synaptic cleft (Zikich et al., 2008). Mice lacking Unc13a (Munc13-1) show impaired glutamatergic neurotransmission and structurally altered neuromuscular junctions (Varoqueaux et al., 2005).

The rs12608932 SNP in *UNC13A* gene is in linkage disequilibrium (LD) with other two SNPs, rs12973192 and rs56041637 (a CATC-repeat insertion), all mapping within intron 20–21 (Ma et al., 2022). Recently, a cryptic exon containing a premature stop codon and whose inclusion is prevented by TDP-43 splicing activity was identified in this intron (Brown et al., 2022; Ma et al., 2022). In motor and cortical-like excitatory neurons derived from induced pluripotent stem cells (iPSCs), the depletion of nuclear TDP-43 led to this cryptic exon inclusion, and, consequently, to a reduced UNC13A protein synthesis due to mRNA nonsense-mediated decay (Brown et al., 2022; Ma et al., 2022). Also in brain samples of patients affected by frontotemporal lobar degeneration with TDP-43 proteinopathy (FTLD-TDP) the inclusion of the *UNC13A* cryptic exon was observed (Ma et al., 2022) together with reduced UNC13A protein level (Brown et al., 2022). Furthermore, the combination of RNA-seq, SNP genotyping and real-time PCR data from post-mortem brain tissues of FTD and ALS patients revealed an association between the top risk variants of *UNC13A* and increased expression of the cryptic exon-containing transcripts (Brown et al., 2022; Ma et al., 2022). Also, in HEK293T cells knocked-down for TDP-43, the three *UNC13A* SNPs (rs12608932, rs56041637 and mainly rs12973192) correlated with an increased inclusion of *UNC13A* cryptic exon due to a decreased TDP-43 binding affinity to the SNP-containing RNA sequence (Ma et al., 2022). This effect was rescued by the expression of TDP-43, thus suggesting a mechanistic link between *UNC13A* risk alleles and TDP-43 splicing activity in ALS/FTD pathophysiology (Ma et al., 2022).

The role of *UNC13A* as a disease modifier in ALS has been broadly explored in the last decade. The rs12608932 minor allele C, the risk allele responsible for increased disease susceptibility (van Es et al., 2009; Diekstra et al., 2012; The ALSGEN Consortium, 2013; Vidal-Taboada et al., 2015; Yang et al., 2019), has been associated also with reduced survival (Diekstra et al., 2012; Chiò et al., 2013; Vidal-Taboada et al., 2015; van Rheenen et al., 2016; Yang et al., 2019; Tan et al., 2020; Ma et al., 2022), which was significantly increased by the treatment with lithium carbonate within three randomized clinical trials (van Eijk et al., 2017). The impact of the rs12608932 SNP on survival after ALS, but not FTD, onset was confirmed also in carriers of pathogenic repeat expansions in the *C9orf72* gene (van Blitterswijk et al., 2014). In our work, we have confirmed the association of the C minor allele with reduced survival of ALS patients. Indeed, the median survival of patients harboring at least one copy of the minor allele of rs12608932 is almost 1 year shorter than homozygotes for the major one. This difference remained significant even after adjusting for possible confounding factors, including age at onset, *C9orf72* mutational status, gender, and site of onset. However, when we ran log-rank pairwise comparisons in the Kaplan–Meier survival analysis to determine which genotypes had different survival distributions under the additive model, we found a significant difference in survival distributions only between the AC vs. AA carriers, but not between the CC vs. AA carriers. Since the 95% CI and the standard error of median survival of CC carriers, who were a minority (11.2%), were far higher than in the other genotypes, it is possible that the results of the *post hoc* analysis might have been influenced by the elevated rate of variability of estimated survival within this group of patients.

An association between the rs12608932 risk allele C and higher age at onset, more frequent bulbar onset and reduced forced vital capacity at diagnosis has been reported in ALS (The ALSGEN Consortium, 2013; Tan et al., 2020), albeit inconsistently (Diekstra et al., 2012; Chen et al., 2014; Vidal-Taboada et al., 2015). According to the ALSFRS-R, patients who were homozygous for the major allele (AA) showed slower progression of symptoms compared to the other genotypes (Vidal-Taboada et al., 2015). In subsequent works, however, either the homozygotes for the major allele (AA) showed the lowest ALSFRS-R compared to the other rs12608932 genotypes (Placek et al., 2019), or no association between the ALSFRS-R and the rs12608932 was found at all (Tan et al., 2020), so that the definite association with this clinical parameter is not clear. Unlike previous works, however, we detected no association of the minor allele C with higher age at onset, bulbar onset and ALSFRS-R, as well as with the presence of clinical LMN signs assessed by LMNS and MRC total score.

Conversely, we found a previously undescribed association of rs12608932 with UMN involvement. Specifically, we showed that patients who are homozygous for the minor allele (CC genotype) have a significantly reduced burden of UMN signs compared to the carriers of the two other possible genotypes, especially compared to those homozygous for the major allele (AA genotype), thus suggesting a “protective” role of the minor C allele against UMN involvement. Noteworthy, the presence of the minor allele, which is associated with reduced survival, does not correlate with the UMN involvement. This finding is in line with current literature, according to which the UMN scores are scarcely associated with survival (Devine et al., 2016).

In fact, the assessment of UMN involvement in ALS might be tricky, due to the simultaneous coexistence of muscle atrophy and LMN signs (Huynh et al., 2016). While the investigation of LMN degeneration is strongly supported by neurophysiological findings, evaluation of UMN involvement mostly relies on clinical clues. In this scenario, the PUMNS has proved to be an efficient, easy and reliable tool to quantify the burden of UMN deterioration in ALS (Quinn et al., 2020). In addition to that, different neurophysiological and imaging biomarkers of corticomotoneuronal pathology have been developed in the last decades, including the increased susceptibility skewness of the precentral cortex through quantitative susceptibility mapping algorithms, which showed a significant correlation with PUMNS (Contarino et al., 2020). Therefore, future studies should extend our genotype–phenotype analysis to additional variables derived from an extensive electrophysiological and neuroimaging assessment, which might take into account early features of motor neuron degeneration.

To date, few works have instead assessed the role of *UNC13A* risk allele on modulating behavioral and cognitive profiles in ALS (Placek et al., 2019; Tan et al., 2020). The rs12608932 minor allele has been associated with imaging, neuropsychological and pathological markers of FTD in ALS, including: (i) significantly reduced cortical thickness in dorsal/ventromedial prefrontal, and anterior/middle temporal areas (Placek et al., 2019; Tan et al., 2020); (ii) lower scores at the reverse digit span test, which mainly evaluates working memory, and at the ECAS, especially concerning the language subdomain (Placek et al., 2019; Tan et al., 2020); (iii) higher rates of behavioral disturbances (Tan et al., 2020); (iv) widespread TDP-43 pathology in middle frontal/temporal and motor cortex (Placek et al., 2019). Here, in our ALS cohort, we did not replicate previous findings regarding the correlation between rs12608932 and the performance at ECAS (total, ALS-specific and ALS non-specific scores). Furthermore, Tan and colleagues demonstrated a higher prevalence of patients classified as ALS-bi and as ALS-FTD, as well as an higher frequency of disinhibition in carriers of the minor allele C (Tan et al., 2020). Within our cohort, instead, we did not detect a different distribution of patients classified according to the Strong revised criteria or a different prevalence of ALS/FTD patients amongst the rs12608932 genotypes under any of the genetic models considered. Further, no significant differences in behavioral symptoms, recorded through the ECAS carer interview and the FBI, emerged.

We recognize that our work has some limitations, including the fact that employing pairwise deletions to handle missing data might have resulted in biased estimates. However, we must point out that the proportion of missing data within our cohort was relatively low. In addition, the ECAS, ECAS Carer Interview and FBI scales were administered only to a minority of patients.

Overall, our findings confirm that *UNC13A* plays a key role in influencing survival after ALS onset and, for the first time, reveal a strong correlation of this locus with UMN involvement. The identification of disease modifiers in ALS is pivotal. In this scenario, an extensive phenotyping of patients, achieved by the simultaneous employment of clinical scales and neuroimaging, neurophysiological and laboratory findings, properly correlated with genotype data, might be helpful in the definition of ALS subgroups, which should guide the follow-up of patients and, hopefully, future clinical trials design.

## Data availability statement

The datasets presented in this study can be found in online repositories. The names of the repository/repositories and accession number(s) can be found at: www.zenodo.org, 10.5281/zenodo.7128674.

## Ethics statement

The studies involving human participants were reviewed and approved by IRCCS Istituto Auxologico Italiano. The patients/participants provided their written informed consent to participate in this study.

## Author contributions

AMan, AR, AB, and NT: conceptualization. AMan, VC, AB, DG, SP, SI, and NT: methodology. AMan, AB, AMar, and NT: formal analysis. AMan, AMar, SM, FV, BP, CM, and NT: investigation. AR, IF, VS, and NT: resources. AMan, AB, AMar, FG, EC, and NT: data curation. AMan: writing – original draft preparation. AMan, AR, and NT: writing – review and editing. AR, VS, and NT: supervision. AR and NT: project administration. VS, AR, and NT: funding acquisition. All authors contributed to the article and approved the submitted version.

## Funding

This work was financially supported by the Italian Ministry of Health (Ricerca Corrente to IRCCS Istituto Auxologico Italiano).

## Conflict of interest

VS received compensation for consulting services and/or speaking activities from AveXis, Cytokinetics, Italfarmaco, Liquidweb Srl, Novartis Pharma AG, and Zambon. He is on the Editorial Board of Amyotrophic Lateral Sclerosis and Frontotemporal Degeneration, European Neurology, American Journal of Neurodegenerative Diseases, Frontiers in Neurology, and Exploration of Neuroprotective Therapy. FV and CM are Review Editor of Frontiers in Aging Neuroscience. BP received compensation for consulting services and/or speaking activities from Liquidweb S.r.l. She is Associate Editor for Frontiers in Neuroscience. NT received compensation for consulting services from Amylyx Pharmaceuticals and Zambon Biotech SA. He is Associate Editor for Frontiers in Aging Neuroscience.

The remaining authors declare that the research was conducted in the absence of any commercial or financial relationships that could be construed as a potential conflict of interest.

## Publisher’s note

All claims expressed in this article are solely those of the authors and do not necessarily represent those of their affiliated organizations, or those of the publisher, the editors and the reviewers. Any product that may be evaluated in this article, or claim that may be made by its manufacturer, is not guaranteed or endorsed by the publisher.
