# Supplementary material for: Association of the risk factor UNC13A with survival and upper motor neuron involvement in amyotrophic lateral sclerosis
Source: Front Aging Neurosci. 2023 Feb 1;15:1067954. doi: 10.3389/fnagi.2023.1067954 (PMC9931189; doi:10.3389/fnagi.2023.1067954)
Supplement: Supplementary file 1 [file Table_1.DOCX]

**Supplementary Table 1** – Multivariate Cox regression models including either the additive or dominant model for the rs12608932 SNP, age at onset, *C9orf72* mutational status, gender, and site of onset (B = unstandardized regression coefficient; SE B = standard error of the coefficient; Exp(B) = hazard ratio). *P* values <0.05 are reported in bold.

|  |  |  |  | **95% CI** | |  |
| --- | --- | --- | --- | --- | --- | --- |
|  | **B** | **SE B** | **Exp(B)** | **LL** | **UL** | ***p* value** |
| ***UNC13A* (rs12608932) – additive model** | 0.219 | 0.078 | 1.245 | 1.069 | 1.450 | **0.005** |
| **AAOO** | 0.043 | 0.005 | 1.044 | 1.034 | 1.054 | **<0.001** |
| ***C9orf72* pathogenic repeat expansion** | -0.810 | 0.243 | 0.445 | 0.277 | 0.716 | **<0.001** |
| **Gender** | -0.161 | 0.110 | 0.852 | 0.686 | 1.056 | 0.144 |
| **Site of onset** | 0.140 | 0.119 | 1.150 | 0.911 | 1.453 | 0.240 |
|  |  |  |  |  |  |  |
|  | **B** | **SE B** | **Exp(B)** | **LL** | **UL** | ***p* value** |
| ***UNC13A* (rs12608932) – dominant model** | 0.335 | 0.106 | 1.398 | 1.135 | 1.721 | **0.002** |
| **AAOO** | 0.042 | 0.005 | 1.043 | 1.034 | 1.053 | **<0.001** |
| ***C9orf72* pathogenic repeat expansion** | -0.784 | 0.242 | 0.457 | 0.284 | 0.735 | **0.001** |
| **Gender** | 0.172 | 0.110 | 0.842 | 0.678 | 1.045 | 0.118 |
| **Site of onset** | 0.156 | 0.120 | 1.169 | 0.925 | 1.479 | 0.192 |

CI: confidence interval; LL: lower limit; UL: upper limit; AAOO: age at onset.

**Supplementary Table 2** – Comparison of PUMNS amongst the *UNC13A* rs12608932 genotypes under an additive, dominant and recessive model in the cohort of ALS patients. The values which were statistically different are reported in bold.

| **Variable** | **AA genotype** | **AC genotype** | **CC genotype** | **(AC + CC) genotype** | **(AA + AC) genotype** | **Additive model (AA vs. AC vs. CC)** | | **Dominant model [AA vs.**  **(AC + CC)]** | **Recessive model [(AA + AC) vs. CC]** |
| --- | --- | --- | --- | --- | --- | --- | --- | --- | --- |
|  | **Median (IQR)** | **Median (IQR)** | **Median (IQR)** | **Median (IQR)** | **Median (IQR)** | |  |  |  |
| **PUMNS** | **10 (4–16)** | 9 (4–16) | **8 (2–13)** | 9 (3–15) | **9 (4–16)** | | **0.044** | 0.172 | **0.015** |

SD: standard deviation; IQR: interquartile range; ALS: amyotrophic lateral sclerosis; PUMNS: Penn Upper Motor Neuron Score.
